# Linkage Mapping Reveals Strong Chiasma Interference in Sockeye Salmon: Implications for Interpreting Genomic Data

**DOI:** 10.1534/g3.115.020222

**Published:** 2015-09-18

**Authors:** Morten T. Limborg, Ryan K. Waples, Fred W. Allendorf, James E. Seeb

**Affiliations:** * School of Aquatic and Fishery Sciences, University of Washington, Seattle, Washington 98195; † National Institute of Aquatic Resources, Technical University of Denmark, Vejlsøvej 39, Silkeborg, Denmark; ‡ Division of Biological Sciences, University of Montana, Missoula, Montana 59812

**Keywords:** gynogenesis, meiosis, recombination, interference, genome data

## Abstract

Meiotic recombination is fundamental for generating new genetic variation and for securing proper disjunction. Further, recombination plays an essential role during the rediploidization process of polyploid-origin genomes because crossovers between pairs of homeologous chromosomes retain duplicated regions. A better understanding of how recombination affects genome evolution is crucial for interpreting genomic data; unfortunately, current knowledge mainly originates from a few model species. Salmonid fishes provide a valuable system for studying the effects of recombination in nonmodel species. Salmonid females generally produce thousands of embryos, providing large families for conducting inheritance studies. Further, salmonid genomes are currently rediploidizing after a whole genome duplication and can serve as models for studying the role of homeologous crossovers on genome evolution. Here, we present a detailed interrogation of recombination patterns in sockeye salmon (*Oncorhynchus nerka*). First, we use RAD sequencing of haploid and diploid gynogenetic families to construct a dense linkage map that includes paralogous loci and location of centromeres. We find a nonrandom distribution of paralogs that mainly cluster in extended regions distally located on 11 different chromosomes, consistent with ongoing homeologous recombination in these regions. We also estimate the strength of interference across each chromosome; results reveal strong interference and crossovers are mostly limited to one per arm. Interference was further shown to continue across centromeres, but metacentric chromosomes generally had at least one crossover on each arm. We discuss the relevance of these findings for both mapping and population genomic studies.

Recombination plays a crucial role in creating novel genetic variation in sexually reproducing species (Barton and Charlesworth 1998; Otto and Lenormand 2002) and for securing proper disjunction of sister chromatids and chromosomes during meiosis. Chiasma interference (hereafter simply referred to as interference) is a fundamental process that influences crossover locations because the formation of a chiasma reduces the chance of a nearby recombination (Sturtevant 1915). Interference was thought to only occur within independent chromosome arms because the centromere acted as a barrier to interference (Mather 1938).

Another important role of recombination includes crossing over between homeologs; these crossovers slow rediploidization of polyploid-origin genomes resulting from recent whole genome duplications (WGD). Pioneering studies considered allozyme loci in lake trout and brook trout hybrids to characterize segregation of duplicated loci in males and presented a meiotic model for explaining this residual tetrasomic inheritance (May *et al.* 1979; Wright *et al.* 1983). These meiotic models were recently synthesized to further promote awareness of these processes (Allendorf *et al.* 2015; May and Delany 2015). Indeed, WGDs have played an important role in the evolution of many polyploid taxa that often serve as complex, but enlightening, models for understanding genome evolution (Comai 2005; Parisod *et al.* 2010; Mable 2013). Knowledge about how meiotic recombination affects the evolution and structure of genomes is crucial to better understand and interpret genomic data.

Historically, basic meiotic processes have mainly been described in model organisms, but today advances in sequencing technology have catalyzed dense genome mapping in nonmodel species. Linkage maps have informed population-based studies, including many of polyploid-origin taxa. However, detailed interrogations of recombination patterns in nonmodel species still lag behind surveys from a few select model organisms (Broman and Weber 2000; Basu-Roy *et al.* 2013). Insights on recombination patterns from a broader range of taxa, including polyploid-origin species, are warranted.

Understanding how recombination shapes genetic diversity across genomes is also crucial for interpreting signals of population divergence in genomic data. The number and distribution of so-called islands of elevated divergence have received immense attention in recent literature on ecological speciation (Ellegren *et al.* 2012; Jones *et al.* 2012; Tine *et al.* 2014). Recombination rates have been invoked as an important factor explaining the size and genomic location of such islands (Renaut *et al.* 2013; Cruickshank and Hahn 2014). A better understanding of how recombination mechanisms vary across a genome will help explain the relative contribution of neutral drift *vs.* divergent selection in creating local regions of elevated divergence among ecotypes or populations within a species (see also discussion in Roesti *et al.* 2013).

Salmonid fishes are an excellent system for studying the role of recombination in genome evolution for a number of reasons. Single pair matings produce thousands of embryos, enabling the examination of large numbers of meiosis from a single individual. Also, details of ploidy manipulation are well worked out; the use of haploid or gynogenetic diploid families greatly enhances genotyping and mapping capabilities (Komen and Thorgaard 2007).

Early inheritance studies in salmonids, although often based on fewer than 50 allozyme loci, suggested strong crossover interference: in females, extended regions between telomeric loci and the centromeres invariably had a single crossover (Thorgaard *et al.* 1983; Allendorf *et al.* 1986; Lindner *et al.* 2000). These early findings were restricted to only a subset of chromosomes and did not include genome-wide interrogations of interference.

We know that homolog recognition and pairing initiates at the telomeres (reviewed in Calderon *et al.* 2014) and interference occurs across centromeres in humans (Colombo and Jones 1997; Broman and Weber 2000) and zebrafish *(Danio rerio*) (Demarest *et al.* 2011). However, detailed genome-wide descriptions of interference are scarce for nonmodel organisms, and the few that exist reveal significant interspecific differences (Segura *et al.* 2013).

Further, the salmonid ancestor went through a recent WGD (Ohno 1970; Allendorf and Thorgaard 1984), and the rediploidization process is not complete. Early inheritance studies in salmonids described a complicated pattern of both disomic and residual tetrasomic inheritance for a suite of isoloci duplicated genes that share alleles (May *et al.* 1979; Wright *et al.* 1983; Allendorf and Danzmann 1997). These isoloci were found to reside mainly in telomeric regions (Thorgaard *et al.* 1983; Allendorf *et al.* 1986; Seeb and Seeb 1986). More recently, studies in a few Pacific salmonid species have combined genotyping by sequencing and the use of haploid mapping to map isoloci (*Oncorhynchus tshawytscha*, Brieuc *et al.* 2014; *O. kisutch*, Kodama *et al.* 2014; *O. keta*, Waples *et al.* 2015). Results suggest that eight syntenic pairs of homeologous chromosome arms remain duplicated across species. These findings are further supported by insights from the rainbow trout (*O. mykiss*) genome sequence that shows conserved sequence identity and gene order between paired paralogous regions (Berthelot *et al.* 2014). Data from additional species will contribute to a more complete understanding of how the WGD has shaped genome evolution in salmonids and other polyploid-origin species.

Our objective is to use genetic mapping to improve understanding of recombination and interference. We combine the use of genotyping by sequencing data from gynogenetic haploid and gynogenetic diploid progeny from a single female sockeye salmon (*O. nerka*). We then: (1) produce a dense genetic map and locate the centromeres and retained duplications; (2) test for the occurrence and strength of interference; and (3) test for the occurrence of interference across centromeres.

## Materials and Methods

### Gynogenetic mapping families

**Figure 1:**
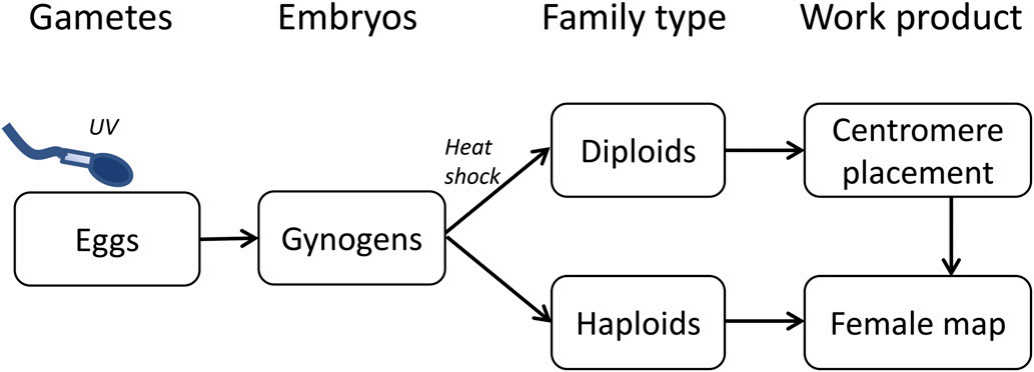
Schematic overview of the construction of gynogenetic families used for linkage map construction. Gametes were collected from a single set of parents followed by UV irradiation of sperm ensuring that only maternal DNA was incorporated into developing embryos. After fertilization, half the gynogens were allowed to develop into gynogenetic haploids and the others were heat-shocked to produce gynogenetic diploids (Chourrout 1980). Both families were RAD sequenced for linkage map construction and recombination analyses.

We produced two families of gynogenetic progeny as described in Chourrout (1980). One gravid female and one male sockeye salmon from the Lake Sammamish population were stripped for eggs and sperm at the Issaquah State Salmon Hatchery (Washington, USA) in December 2012. Fin clips were taken from both parents and stored in ethanol. All progeny were produced by fertilizing all of the eggs with sperm that had been genetically inactivated with 10 min exposure to UV light (Figure 1). A haploid family for linkage map construction was created by placing half of these haploid embryos into the incubator with no further treatment (Figure 1). We produced a second family of gynogenetic diploids (half-tetrads) by exposing the remaining embryos to 10 min heat shock at 28° to induce retention of the second polar body (Figure 1). The first embryo hatched after 86 d; remaining embryos from both families were immediately sampled and stored in ethanol.

DNA was extracted from fin clips and embryos using DNEasy-96 kits (Qiagen, Valencia, CA, USA) following the manufacturer’s directions. Whole embryos were dissected from the yolk and chorion and added directly to the lysis buffer. Both parents as well as 142 putative haploid and 141 putative gynogenetic diploid progeny were genotyped for 96 EST-derived SNPs using 5′-nuclease assays (Elfstrom *et al.* 2006; Storer *et al.* 2012). Genotypes were called using the BioMark software v3.0.2 (Fluidigm, South San Francisco, CA). Embryos with paternal alleles signaled failure of the UV treatment and were excluded from further analysis.

Success of the heat shock treatment was evaluated by screening the same 96 SNPs from the 5′-nuclease assays in putative gynogenetic diploids for the occurrence of completely homozygous embryos. Fully homozygous embryos, for loci segregating in the female parent, would signal failure of the heat shock to incorporate the second polar body; those individuals were also excluded. Genotypes for the 5′-nuclease assay SNPs that were segregating in the gynogenetic families were also used for mapping after filtering for Mendelian inheritance and segregation distortion (see below).

### Sequencing

Genotyping was performed by sequencing restriction site–associated DNA (RAD-seq). All sequencing was done at the University of Oregon High Throughput Sequencing Facility using an Illumina HiSeq2000. We generated sequencing libraries using the restriction enzyme *SbfI* following methods previously described (Baird *et al.* 2008; Everett *et al.* 2012). We ligated unique barcodes (6 bp) to digested DNA following the work of Miller *et al.* (2012). The female parent was sequenced together with gynogenetic haploid and diploid progeny to 101 bp reads (Figure 1). Samples were sequenced on five lanes: two lanes each included the female parent and 47 gynogenetic haploids and three lanes each included 32 gynogenetic diploid progeny.

### Detection and genotyping of polymorphisms

We used the *Stacks* software package v1.04 (Catchen *et al.* 2013) to identify polymorphic loci and to assign genotypes. We used the *process radtags* program to remove reads characterized by low-quality, uncalled bases, or with ambiguous barcodes. Retained reads were demultiplexed and trimmed to 94 bp by removing the barcode and terminal base.

We then identified duplicated loci based on the segregation patterns of alleles in the offspring following the approach of Waples *et al.* (2015). More details about this approach are given in Supporting Information, File S1. Duplicated loci contain sequences from at least two distinct genomic locations and have segregation patterns deviating from disomic Mendelian inheritance (*i.e.*, confounded loci *sensu*; Waples *et al.* 2015).

RAD loci, including duplicated loci, were then genotyped for the haploid family and combined with genotypes for the 5′-nuclease SNPs for linkage map construction. Strict segregation distortion tests served to detect and discard any mis-specified loci. We excluded progeny with less than 0.5 × 10^6^ reads because genotypes could not be reliably assigned with lower sequence coverage.

### Linkage map construction

The haploid family was used to construct the linkage map and the gynogenetic diploid family was used to locate centromeres (Figure 1).

The map was generated by using the minimum spanning tree method implemented in the program MSTMap (Wu *et al.* 2008). Duplicated loci were mapped following the work of Waples *et al.* (2015). We compared our map with previously published linkage maps for sockeye salmon (Everett *et al.* 2012) and rainbow trout (Miller *et al.* 2012) to aid construction and annotation of linkage groups (LGs). We name LGs by considering the numbers given by Everett *et al.* (2012) preceded by a ‘So’ prescript (see File S1 and Table S1 for more details).

Half-tetrad analysis of the gynogenetic diploid offspring was used to place centromeres. Raw Illumina reads were processed in *Stacks* as described for haploids. Only haplotypes for nonduplicated loci were exported. The need to accurately call heterozygote genotypes in the diploid progeny necessitated a higher cut-off threshold of 1.5 × 10^6^ reads compared to the haploid progeny. We exported haplotype alleles from *Stacks* to call genotypes and merged these with genotypes for SNPs detected with the 5′-nuclease assays. Loci with >10% missing genotypes were discarded.

We estimated the frequency of second division segregation (*y*) for each locus following the work of Thorgaard *et al.* (1983) to identify centromeric regions on each LG. Values of *y* are scored as observed heterozygosity in the gynogenetic diploid family and range between zero and one. A heterozygote genotype indicates that an odd number of recombination events have occurred between the locus and the centromere; a homozygous genotype indicates zero or an even number of recombination events. Centromeres are nonrecombining regions that occur as a single location on a linkage group; therefore, adjacent loci are expected to have low *y* values because recombinations will be rare over the short locus-centromere distances. For loci with low *y* values, it can be difficult to identify their orientation in relation to the centromere. Therefore, centromeric regions were conservatively defined as the shortest interval on an LG including all markers with *y* < 0.10.

Finally, loci were assigned to specific chromosomal regions, arms or centromeres, to complete the linkage map and to enable downstream analyses of interference. We labeled loci with *y* < 0.10 as centromeric (c), and, for acrocentric chromosomes, loci with *y* > 0.10 were assigned to reside on arm a1. For metacentric chromosomes, loci with *y* > 0.10 were arbitrarily assigned to reside on arm a1 if located before the centromeric region [*i.e.*, smaller centimorgan (cM) value on the map], or to reside on arm a2 if located after the centromeric region. Finally, it is important to note that even with a few thousand loci, our coverage of the genome will often be insufficient to detect the short p arm for some acrocentric chromosomes.

### Interference

**Figure 2:**
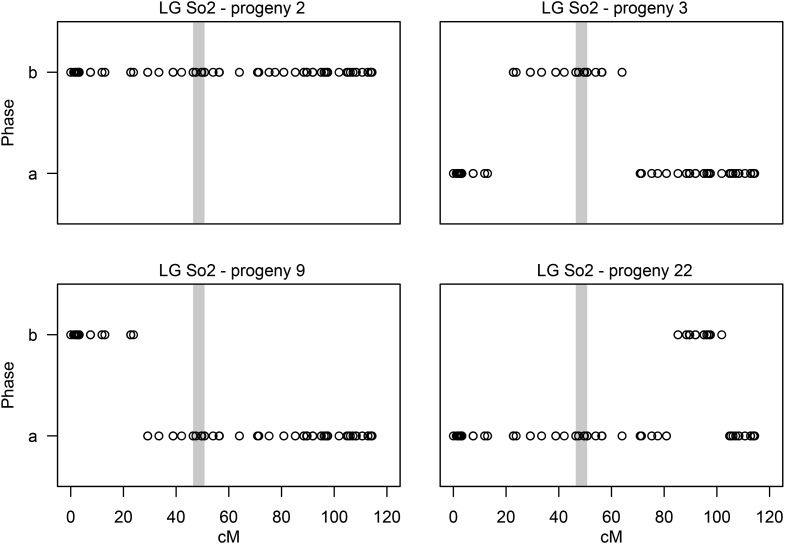
Examples of crossover patterns for four different gynogenetic haploids along LG So2. The two maternal allelic phases are shown on the y-axis. The gray area identifies the centromeric region (see text). Upper left plot shows a haploid offspring with no recombination events, an occurrence in approximately 50% of meiotic products when all four tetrads are sampled. The upper right shows an offspring with one crossover on each arm. The lower left plot depicts an offspring with one crossover on arm a1. The lower right plot shows an example of a haploid offspring with two crossovers on arm a2.

First, we identified recombination events in both haploid and diploid gynogenetic families along each linkage group. We infer phase changes within haploid offspring to be the result of recombination using the parental phase inferred during linkage map construction. We count offspring with zero, one, or two crossover events per arm. In gynogenetic diploids, homozygotes and heterozygotes reflect different phases of the recombinant chromatid resulting from meiosis I. Accordingly, homozygotes and heterozygotes represent distinct maternal phases. Duplicated loci were not considered because alleles could not be assigned to a unique map location. This framework was then used to count number and location of recombination events within each offspring and for each LG (Figure 2). Crossover events were detected as phase changes observed using a sliding window that recorded the mean phase over 11 consecutive marker locations. Up to two crossovers were placed along each chromosome arm. Because double crossover events were rare, we discarded putative third crossovers because these are likely to be the result of genotyping error at one or a few terminal loci (Brieuc *et al.* 2014).

We estimated the genome-wide distribution of *y* to assess the potential occurrence of interference. With no interference, and therefore fully independent crossover locations, the maximum expected value of *y* is 0.67 (Anderson 1925). In contrast, with complete interference, *y* is expected to reach 1.00 at distal locations because there will always be exactly one crossover between distal loci and the centromere.

Next, we estimated the strength of interference by fitting a gamma model to the distribution of observed interchiasma distances using CODA (Gauthier *et al.* 2011). The shape parameter (ν) of the fitted gamma distribution is referred to as the interference parameter; values between zero and one indicate negative interference (*i.e.*, crossover locations are more tightly clustered than expected at random). A value of one signals no interference and that crossover locations are independent of each other (*i.e.*, follow an exponential distribution), and values above one signal positive interference, with higher values indicating stronger interference. Interchiasma distances were only recorded for the diploid progeny to avoid any potential bias from merging observations with the haploid progeny that were used to generate the map distances. The distribution of interchiasma distances was then fit using the biologically realistic two parameter model that allows some fraction (*p*) of observed crossovers to follow a noninterfering pathway (Falque *et al.* 2009; Gauthier *et al.* 2011; Basu-Roy *et al.* 2013). As recommended by Gauthier *et al.* (2011), we used the hill-climbing algorithm to search a parameter space with ν = [1:20] and *p* = [0:1] to find the best fit for each LG. For LGs where estimates of ν equaled 20, the analysis was repeated with increased boundaries. The CIs around estimates of ν were calculated using the Fisher information matrix (Gauthier *et al.* 2011).

### Interference across centromeres

We tested for the occurrence of interference across centromeres on metacentric chromosomes by considering all offspring showing at least one crossover on both arms. We followed the method described in Colombo and Jones (1997) by using the Spearman correlation function:

$$\rho \left(d\right)=\text{corr}(\chi_{\text{a1}},\chi_{\text{a2}}|\max\left(\left[\chi_{\text{a1}},\chi_{\text{a2}}\right]\leq d\right)$$

Here, χ_a1_ and χ_a2_ are the genetic distance from the midpoint of defined centromeric region to the first crossover location on arms a1 and a2. We considered different interval sizes around the centromeric midpoint (*d*) ranging from 10 cM, minimum distance allowing enough observations, and sequentially increasing *d* by 1 cM. A negative correlation indicates interference across centromeres, because a crossover close to the centromere translates into a larger-than-expected distance to the first crossover on the opposite arm. Data were pooled across all metacentric LGs because our data have too few observations to consider individual LGs. We estimated *ρ*(*d*) for both haploid and diploid families; families were treated independently for the reasons given above.

### Data availability

Raw sequence data are deposited in the Short Read Archive (SRA) with accession number SRP063568. Genotypes for both haploid and diploid progeny are deposited on Dryad (doi: 10.5061/dryad.q675s).

## Results

### Linkage map

An average number of 2.5 × 10^6^ reads for 93 haploids and 3.0 × 10^6^ reads for 77 diploids (Table S2) resulted after quality filtering, barcode recovery, demultiplexing, and discarding individuals with low coverage. A total of 3496 loci remained after excluding loci uninformative for mapping (monomorphic in the female parent or with >25% missing genotypes in the offspring). Of these, 868 loci were classified as duplicated. Adding the 31 polymorphic 5′-nuclease SNPs resulted in 3527 loci available for linkage map construction.

**Figure 3:**
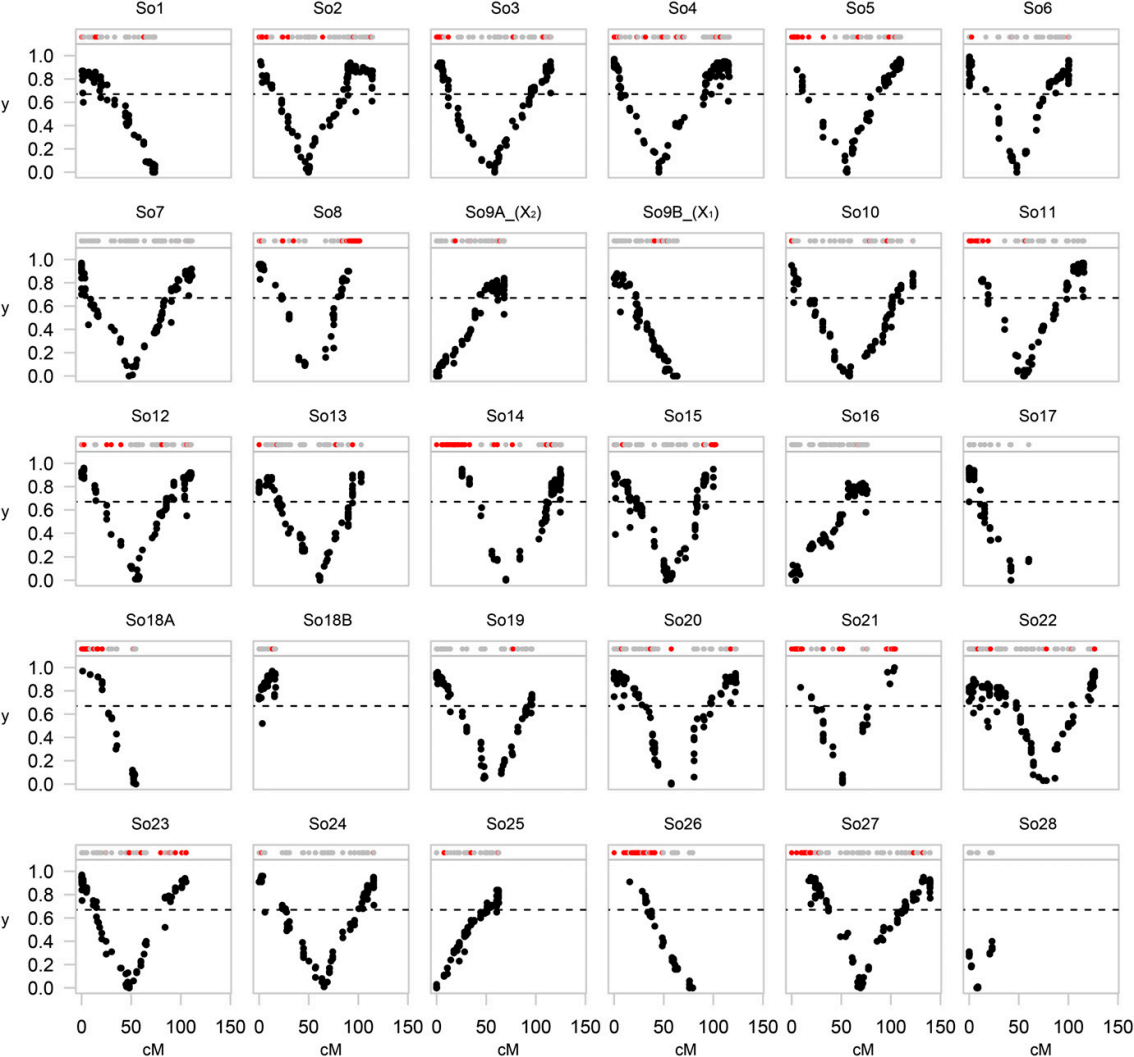
Estimated *y* values plotted along each linkage group. Each plot represents an LG with mapped loci presented by circles in the top with nonduplicated loci shown as gray circles and duplicated loci shown as red circles. Estimated *y* values are plotted on the y-axis for each nonduplicated marker and shown as black circles. The horizontal dotted line equals the maximum expected value of *y* (0.67) without interference. Loci with low *y* values are expected to locate proximal to centromeres, and higher values are expected for loci with increasing distance from centromeres. Overall, these plots serve to effectively distinguish acrocentric (*e.g.*, LG So1) from metacentric (*e.g.*, LG So2) chromosomes and define regions that include the centromere.

Initial linkage group construction using the haploid family produced 30 LGs containing between 21 and 184 markers each (Table S3). Comparison to an existing but less dense map for sockeye salmon served to validate and name LGs (File S1). In two instances, two of our linkage groups matched the same LG in the map of Everett *et al.* (2012); these were denoted by A and B after the LG number. Two LGs corresponded to LG9 in Everett *et al.* (2012) and syntenic comparison with rainbow trout identified these LGs to be the two female sockeye salmon sex chromosomes X_1_ and X_2_ (File S1); these were named So9A_(X_2_) and So9B_(X_1_) following previous descriptions (Thorgaard 1978). For the LGs So18A and So18B pair, So18B does not have a centromere (Figure 3); here, we consider LGs So18A and So18B to be the two arms of a single metacentric chromosome (LG18 in Everett *et al.* 2012). The total map length was 2839 cM and included 2640 nonduplicated and 605 duplicated loci (Table S3).

The gynogenetic diploid family was used to place centromeres; 2562 of the 2640 nonduplicated loci on the linkage map were successfully genotyped for the diploid progeny. Centromeric regions were located by plotting *y* values along the linkage map (Figure 3). Acrocentric chromosomes were characterized by a linear pattern of *y* along linkage groups (*e.g.*, So1), and clearly distinguishable from metacentric chromosomes characterized by a V-shaped *y* plot (*e.g.*, So3) with values below 0.10 defining centromeric regions. After centromere placement, the 30 LGs (2n = 60) translated into a total of 102 chromosome arms (NF = 102; Table S3) which is within the range of previously reported karyotypes for sockeye salmon (NF = 100–104) (Thorgaard 1978; Phillips and Rab 2001).

### Distribution of retained duplicated regions

Duplicated loci were not randomly distributed across the genome. Mapping of two paralogs from 40 duplicated loci enabled identification of six pairs of homeologous regions located on 11 different LGs (Table 1). The majority of duplicated loci (457, 76%) mapped to these 11 LGs, including both arms on So21. Within these 11 LGs, duplicated loci represented between 22 and 66% of all loci and concentrated towards telomeres within distinct arms (red circles in Figure 3; Table S3). Twelve pairs of paralogs colocated within 10 cM on the same LG and likely represent local duplication events that do not originate from the WGD (Waples *et al.* 2015). Another extended, but unpaired, region dominated by duplicated loci occurred on arm a1 of So27; remaining duplicated loci, possibly segmental duplications, were dispersed across the genome (Figure 3).

**Table 1:** Pairing of homeologous chromosome arms in sockeye salmon

Homeolog 1		Homeolog 2		No. of Paired Loci	Homeology in Rainbow Trout
LG	Arm (Region)	LG	Arm (Region)		
So2	a1 (2.9–3.2)	So5	a1 (0.0–6.5)	4	Omy07p-Omy18p
So3	a1 (1.1–5.4)	So14	a1 (5.4–28.8)	3	Omy05p-Omy02p
So8	a2 (92.6–100.1)	So23	a2 (105.3)	4	Omy21p-Omy15q
So11	a1 (8.6)	So18A	a1 (6.8)	1	NA
So15	a2 (101.1–102.5)	So21	a1 (2.2–3.7)	4	Omy17q-Omy13p*^a^*
So21	a2 (103.7)	So26	a1 (12.0–22.2)	4	NA

The linkage group region including the loci supporting a homeologous relationship is given as the start and end location in cM. The two right columns present syntenic comparisons for rainbow trout as inferred from alignment results (File S1; Table S1) and previous reports of homeologous pairs in rainbow trout (Phillips *et al.* 2006; Kodama *et al.* 2014).

a The synteny between So21 and rainbow trout chromosome *Omy*13p were only supported by syntenic reports in rainbow trout (Table S1).

### Interference along chromosome arms

**Figure 4:**
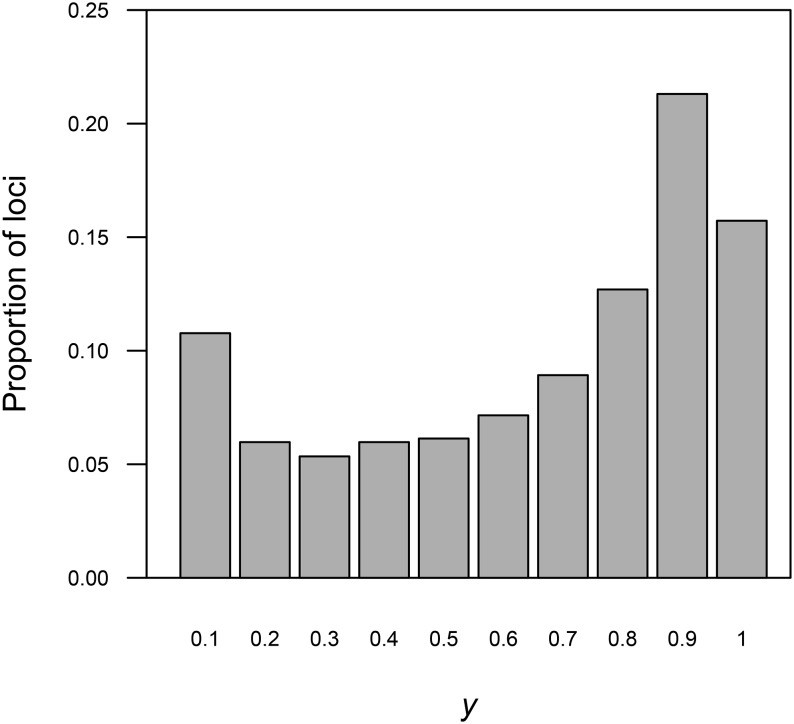
Genome-wide distribution of *y* values estimated for all nonduplicated loci in the gynogenetic diploid family. Loci with values near zero are expected to be proximal to centromeres, and loci with higher values are expected to be more distally located. Values of *y* above 0.67 are only expected with the occurrence of positive crossover interference.

The number of observed recombination events for each arm reveals a pattern of strong, but incomplete, interference. Among haploid progeny, only 64 of 1902 (3.4%) observations showed two crossovers within a single arm (Table 2). Similarly, we observed 178 of 3777 (4.7%) arms with more than one crossover among the diploid progeny (Table 2). Further evidence for interference was revealed by a high fraction of distal loci with *y* values above 0.67 (Figure 4).

**Table 2:** Observed numbers of haploid and diploid gynogenetic offspring with zero, one, or two crossovers are given for each linkage group and for each arm

						**Number of Offspring with 0, 1, or 2 Crossovers**																	
LG	Chromosome Type	Linkage Group Arm Length (cM)				Gynogenetic Haploids									Gynogenetic Diploids								
		Incl. Duplicated Loci		Excl. Duplicated Loci		Entire Linkage Group			Arm 1			Arm 2			Entire Linkage Group			Arm 1			Arm 2		
		Arm 1	Arm 2	Arm 1	Arm 2	0	1	2	0	1	2	0	1	2	0	1	2	0	1	2	0	1	2
So2	M	49	66	47	66	22	47	20	53	40	0	49	34	9	0	2	65	4	73	0	0	66	10
So3	M	58	58	56	58	22	50	20	45	47	1	51	42	0	2	1	65	3	72	2	2	67	6
So4	M	45	71	45	71	22	50	19	67	26	0	45	45	3	1	2	61	4	68	5	1	65	7
So5	M	55	55	50	55	22	47	23	57	36	0	51	40	2	2	13	62	14	63	0	3	74	0
So6	M	48	53	48	53	25	47	19	53	40	0	55	37	1	0	2	70	2	74	1	1	72	3
So7	M	50	61	50	61	20	50	22	57	36	0	55	34	4	3	7	58	10	64	3	3	67	4
So8	M	46	55	46	44	25	52	16	68	25	0	72	21	0	0	8	65	2	73	0	6	68	3
So10	M	54	68	54	68	19	45	24	50	43	0	48	38	7	3	6	60	5	70	1	8	61	7
So11	M	55	61	42	61	22	47	21	52	41	0	48	41	4	1	9	64	14	63	0	1	70	3
So12	M	56	55	56	55	25	43	23	49	44	0	49	42	2	1	2	64	4	69	3	1	68	5
So13	M	61	42	61	42	24	48	19	56	34	3	62	31	0	3	12	48	4	60	9	16	59	1
So14	M	70	56	44	56	26	43	24	64	28	1	67	26	0	2	6	61	9	68	0	2	66	9
So15	M	54	48	54	46	25	50	16	51	39	3	56	37	0	2	5	60	2	65	6	9	68	0
So19	M	56	40	56	40	29	46	18	55	38	0	68	25	0	1	22	53	3	74	0	21	55	1
So20	M	69	54	69	54	19	51	19	59	32	2	59	33	1	0	0	62	0	71	4	0	64	8
So21	M	51	53	42	52	32	38	23	62	31	0	67	26	0	0	19	58	19	58	0	0	77	0
So22	M	76	51	76	51	23	42	25	45	47	1	61	32	0	2	0	59	2	61	12	2	71	2
So23	M	49	57	49	57	20	55	18	54	39	0	43	50	0	3	3	69	3	72	0	7	70	0
So24	M	63	53	63	53	21	47	23	48	44	1	54	38	1	1	1	67	1	74	2	2	69	5
So27	M	72	68	55	68	19	48	24	50	41	2	41	50	2	1	4	66	5	72	0	1	70	5
So28	M	8	15	8	15	74	19	0	85	8	0	82	11	0	37	32	8	55	22	0	51	26	0
So18A	M (arm 1)	53	—	52	—	57	35	0	—	—	—	—	—	—	5	72	0	—	—	—	—	—	—
So18B	M (arm 2)	—	—	—	—	83	9	1	—	—	—	—	—	—	—	—	—	—	—	—	—	—	—
So1	A	69	—	69	—	40	47	6	—	—	—	—	—	—	4	63	6	—	—	—	—	—	—
So16	A	72	—	72	—	56	37	0	—	—	—	—	—	—	6	57	8	—	—	—	—	—	—
So17	A	42	—	42	—	55	38	0	—	—	—	—	—	—	4	58	15	—	—	—	—	—	—
So25	A	63	—	63	—	39	51	3	—	—	—	—	—	—	7	57	10	—	—	—	—	—	—
So26	A	78	—	62	—	46	47	0	—	—	—	—	—	—	8	66	1	—	—	—	—	—	—
So9A_(X2)	A	65	—	65	—	38	52	3	—	—	—	—	—	—	5	56	9	—	—	—	—	—	—
So9B_(X1)	A	57	—	57	—	52	39	2	—	—	—	—	—	—	6	64	2	—	—	—	—	—	—

Chromosome type denotes metacentric (M) or acrocentric (A) chromosomes according to Everett *et al.* (2012) and Figure 3. Lengths of each chromosome arm are given for two linkage map versions; one including and one excluding duplicated loci. Cells with — indicate parameters that could not be estimated for the given LG for reasons given in the text. Linkage groups are ordered by chromosome type to facilitate comparison within and between metacentric and acrocentric chromosomes.

**Figure 5:**
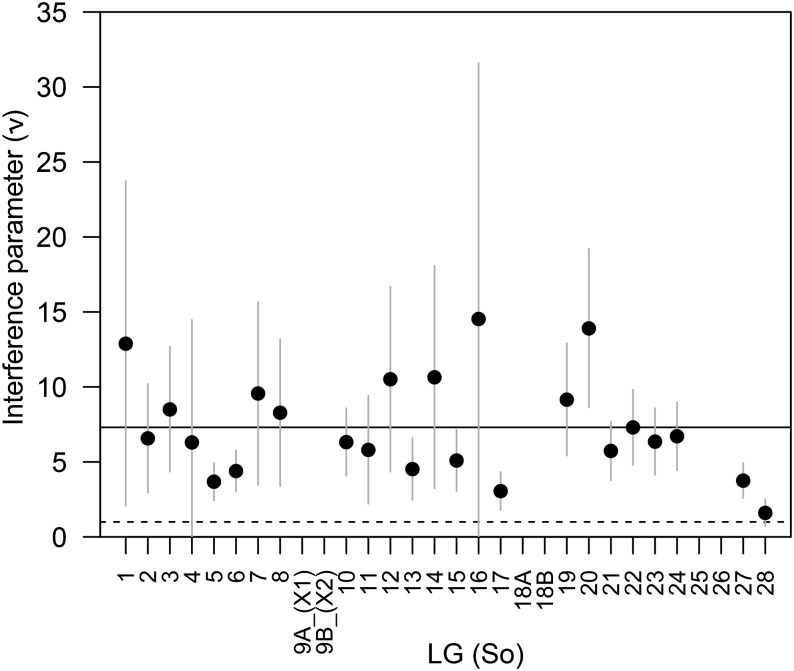
Maximum-likelihood estimates of the interference parameter ν from the gamma model (black circles). Vertical gray lines show the 95% C.I. around each ν estimate. If interference does not affect crossover location, then ν will take a value of 1.0 (horizontal dotted line). The genome-wide average (ν = 7.3) is shown by the solid horizontal line. Estimates of ν were not plotted for six LGs (So9A_(X_2_), So9B_(X_1_), So18A, So18B, So25, and So26) because too few double crossovers on these linkage groups impeded reliable estimation of ν (see text).

We used the observed distribution of interchiasma distances to estimate the strength of interference across chromosomes. Estimates of ν equaled the upper limit of 20 for five LGs (So9A_(X_2_), So9B_(X_1_), So18A, So25, and So26). When the upper limit of ν in the searched parameter space was increased, the ν estimates continued to equal the maximum allowed value (*i.e.*, ν → ∞). Common to these five LGs is that they represent one chromosome arm with few, if any, double crossovers; this likely impeded the fitting of a gamma distribution for the few observed interchiasma distances (Table 2). Therefore, we do not report estimates of ν for these LGs, but conclude that the data signal strong interference as well (Basu-Roy *et al.* 2013; Table S4). The average point estimate among remaining LGs (ν = 7.3) was considerably larger than the value expected without interference (ν = 1). These observations were further supported by low estimates of *p* (*p* = 0.00–0.16) (Table S4) indicating that most crossovers are affected by interference (Falque *et al.* 2009). Figure 5 shows estimates of ν for each LG. All LGs show positive interference, although CIs for So4, So16, and So28 include one, the expected value with no interference. Further, CIs around ν did not include the genome-wide average for seven LGs (So5, So6, So13, So15, So17, So27, So28), suggesting that strength of interference varies among chromosomes (Figure 5). No clear correlation was found when plotting ν as a function of genetic length of each LG (Figure S1).

**Figure 6:**
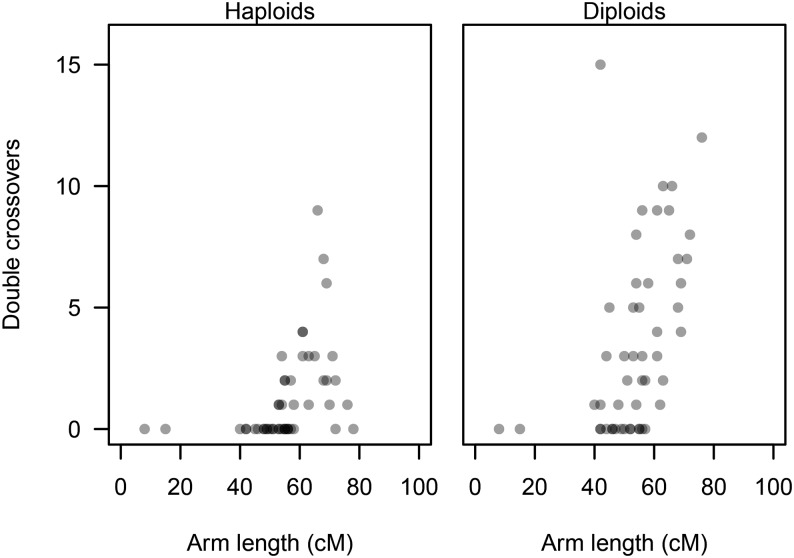
Number of observed double crossovers plotted against arm length. Symbols are shown in transparent gray to highlight over plotting. Results are given for haploid and diploid families separately because observations for diploid offspring do not include duplicated regions.

We detected striking drops of *y* values toward telomeric regions of some LGs; this pattern was particularly pronounced on So2 (arm a2) and So22 (arm a1; Figure 3). These observations result from an increased number of gynogenetic diploid progeny that show two recombinations along these arms (Table 2) because loci located distal to a second chiasma will reduce estimates of *y*. Arm a2 of So2 showed an increased number of double crossovers in the haploids as well, whereas this was not the case for So22 arm a1 (Table 2). In general, occurrence of double crossovers was more common on longer chromosome arms, but absence of double crossovers in some of the longer arms suggest that genetic length is not the only factor determining the frequency of multiple crossovers (Figure 6).

### Interference across centromeres

**Figure 7:**
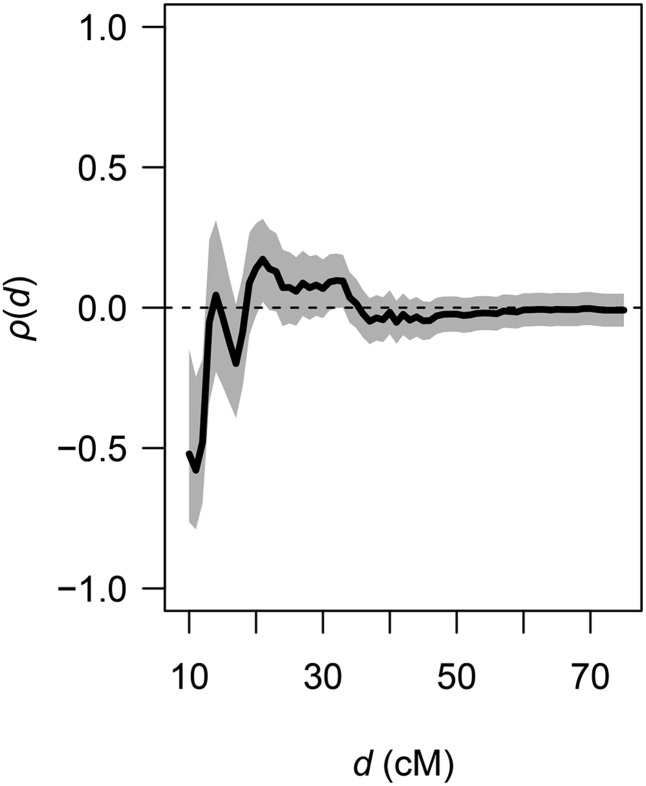
Interference across centromeres. Estimates of the correlation function *ρ*(*d*) = corr(χ_a1_, χ_a2_|max([χ_a1_, χ_a2_] ≤ *d*) for different windows of genetic distance (*d*) in both directions from the midpoint of defined centromeric regions. Estimates of *ρ*(*d*) were obtained for each integer value of *d* ranging from 10 to 75 cM. The gray area denotes the 95% C.I. around *ρ*(*d*). Observations are for all diploid offspring and pooled across all 20 metacentric LGs.

We estimated the Spearman correlation coefficient (*ρ*) between distances from centromeres to the nearest chiasma location on both arms for the 20 metacentric LGs. For the haploid data, no values of *ρ* were significantly different from 0, whereas estimates for diploids were significantly negative for *d* < 15 cM (Figure 7). Nevertheless, the haploid data showed a similar pattern of negative correlations for *d* < 15 cM. Lack of significant correlations in the haploid family may be due to reduced statistical power from the limited number of observations; the similar patterns observed between the haploid and diploid families lead us to conclude that interference does affect crossover patterns across centromeres.

## Discussion

We demonstrate the power of using both haploid and diploid gynogenetic offspring from a single-pair mating to describe detailed patterns of recombination in a duplicated salmonid genome. Results reveal extensive distal regions dominated by duplicated loci as well as strong, but varying, levels of interference for most LGs. Our use of a single female parent comes with some limitations for drawing general conclusions: our data only reflect information from heterozygous loci in the single female parent. Likewise, variability among populations, individuals, and sexes are not captured (*c.f.*, Johnston *et al.* 2015).

### Extensive subtelomeric regions remain duplicated in sockeye salmon

Linkage mapping revealed the occurrence of numerous regions with high levels of retained sequence identity between putative homeologs in sockeye salmon. These observations are consistent with an ongoing rediploidization of the sockeye salmon genome where some homeologous chromosomes still undergo residual tetrasomic inheritance (May *et al.* 1979), a pattern that is shared among many extant species within the salmonid family (Allendorf *et al.* 2015).

We detected six pairs of homeologous regions dominated by duplicated loci as well as an unpaired region on the So27 arm a1. A total of eight conserved pairs of duplicated homeologs have been described for a range of *Oncorhynchus* (Kodama *et al.* 2014) and *Salmo* (Lien *et al.* 2011) species. Although we cannot rule out the possibility that some of these regions have fully diploidized in sockeye salmon based on these data, a more parsimonious explanation is that we have failed to identify another two pairs because we considered too few meiosis (progeny).

Many genomic studies of polyploids, including salmonids, exclude duplicates to avoid inclusion of loci that deviate from standard assumptions such as Hardy-Weinberg proportions (Poland *et al.* 2012; Gagnaire *et al.* 2013; Hyma and Fay 2013; Limborg *et al.* 2014). Consequently, extended regions that remain duplicated are not included in most population genomic studies. The filtering of duplicated loci is especially pertinent to polyploid species where extended telomeric regions are excluded in downstream scans for selection. Important genes located in these duplicated regions will remain undetected, leading to an incomplete understanding of the genetic basis of local adaptation (see discussion in Allendorf *et al.* 2015).

Finally, our study demonstrates how comparative mapping among species represents a powerful tool for validating *de novo* linkage maps in nonmodel species. Indeed, conserved restriction enzyme cut sites among genomes in related species make RAD sequencing, and related techniques, particularly useful for identifying orthologous chromosomal regions through comparative mapping (File S1); a feature that is expected to enrich mapping studies among related species.

### Recombination patterns reveal strong interference

Thorgaard *et al.* (1983) first reported strong interference across the salmonid genome based on data from only 10 allozyme loci, three of which had *y* values at or close to 1.0. We expand the evidence for strong interference by mapping 3245 loci, finding loci with *y* values approaching 1.0 on nearly all linkage groups. It is important to note that we only used nonduplicated loci for estimating *y*. Thus, the proportion of loci with *y* values above 0.67 is likely to be an underrepresentation of the true number (*c.f.*, Lindner *et al.* 2000).

Interestingly, similar to our finding, Lindner *et al.* (2000) also observed an overrepresentation of loci with low values of *y* (see *y* ≤ 0.1 in Figure 4). This peak can be explained by stronger crossover suppression near centromeres, which has been observed in a number of model species (Koehler *et al.* 1996; Lamb *et al.* 2005; Rockmill *et al.* 2006). One explanation involves selection against crossovers proximal to the centromere that has been shown to destabilize meiotic segregation and increase occurrence of nondisjunction (Talbert and Henikoff 2010). Assuming an even genome coverage of loci, our observation of an increased number of loci with low *y* values supports a similar model of selection against crossovers proximal to centromeres in sockeye salmon.

Our study adds new insights about genome-wide interference in a salmonid genome by presenting the first quantitative estimates of the strength of interference across each chromosome. Positive interference for most, if not all, chromosomes described for sockeye salmon in this study is common to a range of other eukaryotes including plants (Lhuissier *et al.* 2007; Giraut *et al.* 2011) and mammals (Broman *et al.* 2002; Segura *et al.* 2013). Nevertheless, most existing data come from model species, and the strength of interference varies greatly among taxa (Giraut *et al.* 2011), illustrating the need for obtaining species-specific estimates. Importantly, we demonstrate that data from mapping studies that use genotyping by sequencing in nonmodel species can be used to obtain quantitative estimates of interference. Because no additional data are needed, we advocate a routine execution of interference analyses in future mapping studies. This will lead to a deeper understanding of variation across more taxa and of how recombination interference is affecting genome evolution in general.

Observations of more than one crossover, coupled with subtelomeric regions showing declining values of *y*, illustrate that interference is not complete, a pattern supported by observations in both haploid and diploid families. The occurrence of double crossovers was generally more common on longer chromosome arms (Figure 6). Our measurement of recombination length, without knowledge of the corresponding physical length, complicates interpretation. It is tempting to posit that interference simply erodes with chromosome arm length, increasing the chance of observing multiple crossovers for longer chromosomes (Broman *et al.* 2002; Giraut *et al.* 2011; Mary *et al.* 2014). However, significant outlier LGs belie this interpretation (see Table 2): LG So17 is a short acrocentric (42 cM), yet it has 15 double crossovers; So14 and So27 have two of the longest arms (≥70 cM), yet they have no double crossovers. Clearly the recombination length of chromosome arms is not the only factor affecting interference, and recombination patterns are likely dictated by a complex blend of different meiotic mechanisms.

Varied strength of interference among chromosomes would have strong implications for interpreting genome data; unfortunately, there are little, if any, data on this issue in nonmodel taxa. Although no clear correlation between strength of interference and genetic distance is observed here, the two shortest LGs, So17 and So28, also have the lowest estimates of ν (Table S4). Evidence from yeast and humans also shows that interference is weaker on small chromosomes (Kaback *et al.* 1992; Kaback 1996). However, our observation of interchromosomal differences has to be interpreted with care. All LGs are shorter than 150 cM and contain few double crossovers that inevitably translate into uncertainties because ν estimates are conditioned on the observation of interchiasma distances (Broman and Weber 2000). Nevertheless, we do detect a genome-wide pattern of strong, but incomplete, interference with some observed variation in the strength of interference among chromosomes; these results have important implications for the analyses of genomic data (see below).

### Interference occurs across centromeres

We found evidence that interference occurs across a region spanning ∼15 cM on either side of the centromere; this implies that crossovers occurring near the centromere affect recombination events on the opposite arm. Transcentromere interference has been reported in other species groups, including insects (Colombo and Jones 1997), mammals (Broman and Weber 2000; Mary *et al.* 2014), and fish (Demarest *et al.* 2011). In our data, the effect declines rapidly with increasing distance from the centromere and disappears at distances greater than 15 cM. This rapid decline of transcentromere interference contrasts with patterns observed in humans (Broman and Weber 2000) and pigs (Mary *et al.* 2014).

### Implications for analyzing genomic data

Recombination is an important evolutionary process that generates novel genetic variation. Compelling theoretical constructs predict an evolutionary advantage of increased recombination rates (Felsenstein 1974; Barton and Charlesworth 1998), and empirical evidence for this theory has been found in, for example, *Escherichia coli* (Cooper 2007) and *Drosophila melanogaster* (Presgraves 2005; Campos *et al.* 2014). Yet, it has proven nontrivial to broadly demonstrate such an effect because studies have been restricted to a few model species and because recombination rates vary among chromosomes and the few taxonomic groups studied (Cutter and Payseur 2013). In this study, we demonstrate how data generated for linkage mapping in nonmodel species can also be used to estimate interference. These methods can be used to estimate species-specific rates of interference whenever a linkage map is created, leading to a more complete understanding of how this fundamental meiotic process shapes genomic data across species.

Our results will also aid interpretation of new analyses facilitated by genomic data. One such method involves examining length distributions of genomic runs of homozygosity (ROH) to infer migration or inbreeding history (Pool and Nielsen 2009; Kirin *et al.* 2010; Harris and Nielsen 2013). The length of the ROH is affected by recombination patterns, because shorter runs are expected in regions with higher recombination rates. Interference is also expected to affect the length distribution of ROHs, especially if the strength of interference varies among the individuals and populations being compared (Broman and Weber 2000). The concrete effect of strong interference on these analyses remains unclear but deserves attention in future developments of these methods. A first step could include estimates of interference within each of the populations being compared; if the strength and pattern of interference are similar among populations, then it will be easier to justify conclusions based on ROH comparisons.

Assumptions of interference strength are crucial to genetic mapping functions. Models assuming different levels of interference have been shown to produce different results based on the same data (Zhao and Speed 1996). Our genome-wide estimate of the interference parameter (ν = 7.3) in sockeye salmon is closer to that assumed by the Carter-Falconer mapping model (ν = 7.6) than the more often used Haldane (ν = 1.0) and Kosambi (ν = 2.6) functions (Zhao and Speed 1996; Broman and Weber 2000). With the accumulation of more accurate estimates of species-specific interference levels it will be possible to improve species-specific mapping efforts; such improvements will have implications when mapping genes that control important phenotypic traits.

## 

## Supplementary Material

Supporting Information
