# Supplementary material for: Linkage Mapping Reveals Strong Chiasma Interference in Sockeye Salmon: Implications for Interpreting Genomic Data
Source: G3 (Bethesda). 2015 Sep 18;5(11):2463–73. doi: 10.1534/g3.115.020222 (PMC4632065; doi:10.1534/g3.115.020222)
Supplement: Supporting Information [file supp_5_11_2463__index.html]

Linkage Mapping Reveals Strong Chiasma Interference in Sockeye Salmon: Implications for Interpreting Genomic Data — Supporting Information 

# Linkage Mapping Reveals Strong Chiasma Interference in Sockeye Salmon: Implications for Interpreting Genomic Data

## Supporting Information for Limborg *et al.*, 2015

**Files in this Data Supplement:**

- Supporting Information - File S1, Figure S1, and Tables S1,S2, and S4 (PDF, 350 KB)
- File S1 - Supporting Materials and Methods (PDF, 137 KB)
- Figure S1 - Estimates of the interference parameter (*ν*). (PDF, 146 KB)
- Table S1 - The table presents syntenic relationships among chromosome arms between linkage maps for sockeye salmon (this study) and rainbow trout (Miller *et al.* 2012). (PDF, 140 KB)
- Table S2 - Number of raw reads and number of reads retained after filtering for each individual. (PDF, 147 KB)
- Table S4 - Interference results from the CODA analysis with estimates and (±95% CI) for *ν* and *p* for all LGs. (PDF, 151 KB)
- Table S3 - Linkage map with the following information for each locus; marker name, supported segregation model inferred from the method of Waples *et al.* (2015), duplication status, LG (So), LG arm, position (cM), *y* value, RAD-tag sequence. (.xlsx, 311 KB)
