# Supplementary material for: Linkage Mapping Reveals Strong Chiasma Interference in Sockeye Salmon: Implications for Interpreting Genomic Data
Source: G3 (Bethesda). 2015 Sep 18;5(11):2463–73. doi: 10.1534/g3.115.020222 (PMC4632065; doi:10.1534/g3.115.020222)
Supplement: Supporting Information [file supp_g3.115.020222_020222SI.pdf]

G3: Genes | Genomes | Genetics

Linkage mapping reveals strong chiasma interference in sockeye salmon: Implications for interpreting genomic data

Morten T. Limborg[*,§], Ryan K. Waples[*], Fred W. Allendorf[†], James E. Seeb[*]

[*]School of Aquatic and Fishery Sciences, University of Washington, 1122 NE Boat Street, Box 355020, Seattle, Washington 98195, USA

[§]National Institute of Aquatic Resources, Technical University of Denmark, Vejlsøvej 39, Silkeborg, Denmark

[†] Division of Biological Sciences, University of Montana, Missoula, MT 59812

Corresponding author: Morten T. Limborg, School of Aquatic and Fishery Sciences, University of Washington, 1122 NE Boat Street, Box 355020, Seattle, Washington 98195, USA, Tel (+45) 61282984, Fax 1-206-685-7471. E-mail: morten.limborg@gmail.com

DOI: 10.1534/g3.115.020222

File S1

Supporting Materials and Methods

Restriction site associated DNA (RAD) sequencing, SNP discovery, and genotyping

We identified and resolved duplicated loci, here defined as assemblies representing two distinct genomic locations, using the allelic segregation patterns in the haploid offspring. In *Stacks* (Catchen *et al.* 2011; Catchen *et al.* 2013) and during other approaches to discover and genotype loci *de novo*, sequences are aligned, and similar sequences are assembled into loci. In cases of sequence, gene, or genome duplication, paralogous sequences can erroneously be assigned to the same locus, here termed duplicated loci. We follow the approach of Waples *et al.* (2015) and attempt to resolve and include individual loci from these confounded assemblies on the linkage map.

For the sockeye salmon (*Oncorhynchus nerka*) parent and each haploid offspring, we considered stacks of minimum two identical reads as representing unique alleles and allowed four unique stacks (the maximum number of expected alleles for a duplicated locus) with up to four nucleotide mismatches for building *de novo* loci. In order to resolve duplicated loci based on segregation within the mapping family, we disabled the deleveraging algorithm within Stacks (Catchen *et al.* 2013), which is designed to resolve them based on sequence similarity. Then we created a catalog from the female parent and assigned genotypes to haploid offspring by matching to RAD loci in the parental catalog.

We identified duplicated loci as *Stacks* assemblies with segregation patterns consistent with two confounded disomic loci, considering the possibility of full allelic identity (i.e. isoloci). For inclusion on a linkage map, a locus must be heterozygous (segregating) in the parent. Identified duplicated loci were resolved into zero, one, or two separate loci eligible for inclusion on the linkage map, based on variability in the female parent and the ability to infer segregation in the offspring (see Waples *et al.* 2015).

At this point, genotypes from 5'-nuclease assays and RAD loci were combined into a single data set for creation of a linkage map. Loci with missing genotypes in more than 25% of the haploid

offspring or that showed significant segregation distortion ($P<0.05$ after a false discovery rate correction) were removed prior to linkage map construction (Waples *et al.* 2015).

Comparative mapping, annotation, and sex chromosomes

Here, we used both intra- and interspecific comparisons to effectively validate LG assemblies, maintain nomenclature of LGs and identify sex chromosomes.

Annotation of linkage groups

We compared the linkage map with the existing male and female maps for anadromous sockeye salmon developed with a similar RAD sequencing approach (Everett *et al.* 2012). We used the BLASTN software (BLAST version 2.2.28; Altschul *et al.* 1990) to align a fasta file with sequences for each locus on our linkage map. The first 59 bp of each sequence were considered to match another fasta file with 59 bp allelic sequences for each locus on the map by Everett *et al.* (2012). We allowed a maximum of two mismatches and considered the alignment with the lowest e-value as signifying homology between the two sockeye salmon maps. Matching RAD loci were used to identify and annotate LGs according to Everett *et al.* (2012).

Identification of sex chromosomes

We aligned sequences for mapped loci to a linkage map for rainbow trout (*O. mykiss*) generated with a similar RAD-sequencing approach (Miller *et al.* 2012) to identify the X_1 and X_2 sex chromosomes based on known synteny. Sockeye salmon have a unique X_1X_2Y sex chromosome system, where a single copy of the metacentric Neo-Y chromosome is present only in males whereas females have two copies each of the two acrocentric chromosomes X_1 and $X_{2.}$ (Thorgaard 1978; Faber-Hammond *et al.* 2012).The sockeye salmon ancestral sex chromosome X_1 is acrocentric and known to share homology with chromosome *Omy*08 in rainbow trout, whereas the acrocentric X_2 share homology with *Omy*02.

The rainbow trout map has been paired with corresponding physical chromosomes for rainbow trout (Palti *et al.* 2011) which allowed for the identification of sockeye salmon linkage groups that share synteny with *Omy*08 and *Omy*02. We used the BLASTN software to align a fasta file with RAD-tag sequences from our map to all 60 bp mapped RAD-tags on the rainbow trout linkage map (Miller *et al.* 2012). Compared to the intraspecific comparison described above, we allowed sequences to be

more diverged between the two species; we allowed up to two mismatches, alignment lengths of at least 58 of 60 bp, and a maximum of two gaps as signifying orthologous loci in rainbow trout. The alignment with the lowest e-value was retained.

The ancestral sex chromosome (X_1) was identified as LG9B and the acrocentric autosome (X_2) was identified as LG9A on our map. This comports with the result that LG9 represents the metacentric Neo-Y chromosome in the male sockeye salmon map of Everett *et al.* (2012). Furthermore, most LGs aligned to either a single or two different LGs between the two species (Suppl. Table S1). Conserved synteny has been observed among chromosome arms coupled with a range of Robertsonian rearrangements after the two species diverged (see discussion in Naish *et al.* 2013). In a few cases, loci from a single LG aligned to more than two LGs in the other species; this may be explained by single erroneous alignments since no more than two LGs were supported by more than one locus from the corresponding sockeye salmon LG (Table S1).

References

Altschul, S. F., W. Gish, W. Miller, E. W. Myers and D. J. Lipman, 1990 Basic local alignment search tool. J. Mol. Biol. 215**:** 403-410.

Catchen, J., P. A. Hohenlohe, S. Bassham, A. Amores and W. A. Cresko, 2013 Stacks: an analysis tool set for population genomics. Mol. Ecol. 22**:** 3124-3140.

Catchen, J. M., A. Amores, P. Hohenlohe, W. Cresko and J. H. Postlethwait, 2011 Stacks: Building and genotyping loci de novo from short-read sequences. G3 1**:** 171-182.

Everett, M. V., M. R. Miller and J. E. Seeb, 2012 Meiotic maps of sockeye salmon derived from massively parallel DNA sequencing. BMC Genomics 13**:** 521.

Faber-Hammond, J., R. B. Phillips and L. K. Park, 2012 The sockeye salmon neo-Y chromosome is a fusion between linkage lroups orthologous to the coho Y chromosome and the long arm of rainbow trout chromosome 2. Cytogenet Genome Res 136**:** 69-74.

Miller, M. R., J. P. Brunelli, P. A. Wheeler, S. X. Liu, C. E. Rexroad *et al.*, 2012 A conserved haplotype controls parallel adaptation in geographically distant salmonid populations. Mol. Ecol. 21**:** 237-249.

Naish, K. A., R. B. Phillips, M. S. O. Brieuc, L. R. Newton, A. E. Elz *et al.*, 2013 Comparative genome mapping between Chinook salmon (*Oncorhynchus tshawytscha*) and rainbow trout (*O. mykiss*) based on homologous microsatellite loci. G3 3**:** 2281-2288.

Palti, Y., C. Genet, M.-C. Luo, A. Charlet, G. Gao *et al.*, 2011 A first generation integrated map of the rainbow trout genome. BMC Genomics 12**:** 180.

Thorgaard, G. H., 1978 Sex-chromosomes in sockeye salmon - Y-Autosome fusion. Can. J. Genet. Cytol. 20**:** 349-354.

Waples, R. K., L. W. Seeb and J. E. Seeb, 2015 Linkage mapping with paralogs exposes regions of residual tetrasomic inheritance in chum salmon (*Oncorhynchus keta*). Mol. Ecol. Resour. doi: 10.1111/1755-0998.12394.

Table S1 The table presents syntenic relationships among chromosome arms between linkage maps for sockeye salmon (this study) and rainbow trout (Miller *et al.* 2012). The number of loci supporting each syntenic pair is given.

Sockeye salmon LG (This study)	Number of loci supporting synteny	Rainbow trout LG (Miller et al. 2012)	Rainbow trout Chr. (Palti et al. 2011)
**1**	5	WS02	Omy22
**1**	1	WS16	Omy27
**2**	4	WS25	Omy07
**3**	1	WS04	Omy09
**3**	2	WS06	Omy03
**4**	8	WS03	Omy05
**4**	2	WS19	Omy18
**5**	1	WS19	Omy18
**5**	4	WS24	Omy25
**6**	1	WS12	Omy15
**7**	3	WS04	Omy09
**7**	1	WS10	Omy14
**7**	1	WS12	Omy15
**8**	1	WS17	Omy24
**8**	1	WS26	Omy21
**9A_(X_2)**	4	WS18	Omy02
**9B_(X_1)**	2	WS05	Omy08
**10**	2	WS14	Omy10
**10**	1	WS24	Omy25
**11**	1	WS03	Omy05
**11**	3	WS16	Omy27
**12**	2	WS04	Omy09
**12**	4	WS24	Omy25
**13**	2	WS01	Omy04
**13**	1	WS06	Omy03
**13**	2	WS07	Omy11

**14**	1	WS17	Omy24
**14**	1	WS18	Omy02
**15**	4	WS23	Omy17
**16**	1	WS15	Omy20
**16**	1	WS20	Omy01
**17**	3	WS01	Omy04
**18A**	0	N/A	N/A
**18B**	1	WS22	Omy19
**19**	3	WS11	OmySex
**20**	3	WS08	Omy16
**20**	1	WS09	Omy28
**20**	1	WS23	Omy17
**21**	0	N/A	N/A
**22**	5	WS07	Omy11
**22**	2	WS21	Omy12
**23**	3	WS08	Omy16
**23**	1	WS12	Omy15
**23**	1	WS24	Omy25
**24**	6	WS03	Omy05
**24**	1	WS05	Omy08
**24**	1	WS18	Omy02
**25**	1	WS14	Omy10
**25**	5	WS20	Omy01
**26**	1	WS08	Omy16
**27**	1	WS13	Omy06
**28**	0	N/A	N/A

Table S2 Number of raw reads and number of reads retained after filtering for each individual. The last column shows whether an individual was discarded from mapping analyses.

Individual ID	Gynogenetic family	Barcode	Total reads	Retained reads	Discarded
SISSAQ12X_0001	Female parent	AAACGG	3510906	2642057	No
SISSAQ12X03H_0008	Haploid	AACGTT	1302693	1059747	No
SISSAQ12X03H_0016	Haploid	AACTGA	701572	514333	No
SISSAQ12X03H_0024	Haploid	AAGACG	2121270	1750342	No
SISSAQ12X03H_0032	Haploid	AAGCTA	4411699	3782943	No
SISSAQ12X03H_0040	Haploid	AATATC	4077910	3449235	No
SISSAQ12X03H_0048	Haploid	AATGAG	1684251	1108657	No
SISSAQ12X03H_0056	Haploid	ACAAGA	1570911	1036513	No
SISSAQ12X03H_0064	Haploid	ACAGCG	1738236	1217951	No
SISSAQ12X03H_0072	Haploid	ACATAC	1830911	1209645	No
SISSAQ12X03H_0080	Haploid	ACCATG	3308379	2340190	No
SISSAQ12X03H_0088	Haploid	ACCCCC	1641003	1117710	No
SISSAQ12X03H_0001	Haploid	ACTCTT	1064767	864668	No
SISSAQ12X03H_0009	Haploid	ACTGGC	1071670	886602	No
SISSAQ12X03H_0017	Haploid	AGCCAT	1631060	1332419	No
SISSAQ12X03H_0025	Haploid	AGCGCA	2641343	2252937	No
SISSAQ12X03H_0033	Haploid	AGGGTC	1882880	1561840	No
SISSAQ12X03H_0041	Haploid	AGGTGT	3832713	3288036	No
SISSAQ12X03H_0049	Haploid	AGTAGG	2315293	1573483	No
SISSAQ12X03H_0057	Haploid	AGTTAA	923301	610959	No
SISSAQ12X03H_0065	Haploid	ATAGTA	2536516	1740342	No
SISSAQ12X03H_0073	Haploid	ATCAAA	2268868	1596472	No
SISSAQ12X03H_0081	Haploid	ATGCAC	1019779	695063	No
SISSAQ12X03H_0089	Haploid	ATGTTG	1887750	1282210	No
SISSAQ12X03H_0002	Haploid	ATTCCG	2031588	1757416	No
SISSAQ12X03H_0010	Haploid	CAAAAA	3197566	2586942	No
SISSAQ12X03H_0018	Haploid	CAATCG	4938520	4212978	No
SISSAQ12X03H_0026	Haploid	CACCTC	2882513	2414350	No
SISSAQ12X03H_0034	Haploid	CAGGCA	2743394	2240740	No
SISSAQ12X03H_0042	Haploid	CATACT	8801363	7171004	No
SISSAQ12X03H_0050	Haploid	CCATTT	5723786	4040689	No
SISSAQ12X03H_0058	Haploid	CCCGGT	3838609	2675535	No
SISSAQ12X03H_0066	Haploid	CCCTAA	4392264	3111974	No
SISSAQ12X03H_0074	Haploid	CCGAGG	6849875	4762105	No
SISSAQ12X03H_0082	Haploid	CCGCAT	2454314	1693534	No
SISSAQ12X03H_0090	Haploid	CCTAAC	953653	609854	No
SISSAQ12X03H_0003	Haploid	CGAGGC	1964897	1665082	No
SISSAQ12X03H_0011	Haploid	CGCAGA	4408219	3803598	No
SISSAQ12X03H_0019	Haploid	CGCGTG	1277376	1054193	No
SISSAQ12X03H_0027	Haploid	CGGTCC	2638079	2230814	No

SISSAQ12X03H_0035	Haploid	CGTCTA	2399842	2050434	No
SISSAQ12X03H_0043	Haploid	CGTGAT	7678099	6396252	No
SISSAQ12X03H_0051	Haploid	CTACAG	2855029	1880754	No
SISSAQ12X03H_0059	Haploid	CTCGCC	2880646	2081839	No
SISSAQ12X03H_0067	Haploid	CTGCGA	3435265	2460981	No
SISSAQ12X03H_0075	Haploid	CTGGTT	3926014	2713102	No
SISSAQ12X03H_0083	Haploid	CTTATG	4548577	3073605	No
SISSAQ12X03H_0091	Haploid	CTTTGC	2347081	1669222	No
SISSAQ12X03H_0004	Haploid	GAAATG	2075891	1741249	No
SISSAQ12X03H_0012	Haploid	GAACCA	1662262	1398563	No
SISSAQ12X03H_0020	Haploid	GACGAC	1723897	1417738	No
SISSAQ12X03H_0028	Haploid	GACTCT	1688297	1382207	No
SISSAQ12X03H_0036	Haploid	GAGAGA	5510082	4717496	No
SISSAQ12X03H_0044	Haploid	GATCGT	7464556	6487418	No
SISSAQ12X03H_0052	Haploid	GCAGAT	2942628	2055403	No
SISSAQ12X03H_0060	Haploid	GCATGG	3401023	2376889	No
SISSAQ12X03H_0068	Haploid	GCCGTA	3812296	2743382	No
SISSAQ12X03H_0076	Haploid	GCGACC	5587118	3999305	No
SISSAQ12X03H_0084	Haploid	GCGCTG	3797363	2661530	No
SISSAQ12X03H_0092	Haploid	GCTCAA	3518028	2525273	No
SISSAQ12X03H_0005	Haploid	GGACTT	1976980	1683825	No
SISSAQ12X03H_0013	Haploid	GGCAAG	3012049	2517255	No
SISSAQ12X03H_0021	Haploid	GGGCGC	1172978	1026354	No
SISSAQ12X03H_0029	Haploid	GGGGCG	1387457	1196799	No
SISSAQ12X03H_0037	Haploid	GGTACA	5969662	5196322	No
SISSAQ12X03H_0053	Haploid	GTAAGT	2449586	1654856	No
SISSAQ12X03H_0061	Haploid	GTATCC	3164107	2226766	No
SISSAQ12X03H_0069	Haploid	GTCATC	4096473	2904037	No
SISSAQ12X03H_0077	Haploid	GTGCCT	3214148	2292191	No
SISSAQ12X03H_0085	Haploid	GTGTAA	3929391	2840228	No
SISSAQ12X03H_0093	Haploid	GTTGGA	4370537	3098093	No
SISSAQ12X03H_0006	Haploid	TAAGCT	2112534	1744767	No
SISSAQ12X03H_0014	Haploid	TAATTC	2175700	1744360	No
SISSAQ12X03H_0022	Haploid	TACACA	2422145	1920864	No
SISSAQ12X03H_0030	Haploid	TACGGG	3297393	2711704	No
SISSAQ12X03H_0038	Haploid	TAGTAT	4507349	3848697	No
SISSAQ12X03H_0046	Haploid	TATCAC	8601558	7343956	No
SISSAQ12X03H_0054	Haploid	TCAAAG	3775470	2573274	No
SISSAQ12X03H_0062	Haploid	TCCTGC	3271158	2307853	No
SISSAQ12X03H_0070	Haploid	TCGATT	4960926	3436170	No
SISSAQ12X03H_0078	Haploid	TCGCCA	4641966	3389129	No
SISSAQ12X03H_0086	Haploid	TCGGAC	3012889	2056463	No
SISSAQ12X03H_0094	Haploid	TCTCGG	4460161	3183675	No
SISSAQ12X03H_0007	Haploid	TCTTCT	1400378	1133952	No

SISSAQ12X03H_0015	Haploid	TGAACC	1995321	1564231	No
SISSAQ12X03H_0023	Haploid	TGACAA	2415156	1980757	No
SISSAQ12X03H_0031	Haploid	TGCCCG	2016378	1659369	No
SISSAQ12X03H_0039	Haploid	TGCTTA	8313218	7099414	No
SISSAQ12X03H_0047	Haploid	TGGGGA	4257482	3620439	No
SISSAQ12X03H_0055	Haploid	TTATGA	3563281	2482462	No
SISSAQ12X03H_0063	Haploid	TTCCGT	2216930	1580704	No
SISSAQ12X03H_0071	Haploid	TTCTAG	2934614	2012744	No
SISSAQ12X03H_0079	Haploid	TTGAGC	4650794	3333142	No
SISSAQ12X03H_0087	Haploid	TTTAAT	2860889	2025342	No
SISSAQ12X03H_0045	Haploid	GGTTTG	212550	72442	Yes
SISSAQ12X03G_0048	Diploid	TGGGGA	601909	46228	Yes
SISSAQ12X03G_0009	Diploid	AACGTT	803942	374549	Yes
SISSAQ12X03G_0049	Diploid	AATGAG	989596	452567	Yes
SISSAQ12X03G_0025	Diploid	AAGACG	1198295	640910	Yes
SISSAQ12X03G_0057	Diploid	ACAAGA	1282985	729717	Yes
SISSAQ12X03G_0058	Diploid	AGTTAA	1275369	742984	Yes
SISSAQ12X03G_0017	Diploid	AACTGA	1645346	855666	Yes
SISSAQ12X03G_0074	Diploid	ATCAAA	1370569	933352	Yes
SISSAQ12X03G_0002	Diploid	ACTCTT	1678815	1038926	Yes
SISSAQ12X03G_0069	Diploid	GCCGTA	1317912	1053602	Yes
SISSAQ12X03G_0037	Diploid	GAGAGA	2044581	1083206	Yes
SISSAQ12X03G_0021	Diploid	GACGAC	1754262	1179654	Yes
SISSAQ12X03G_0026	Diploid	AGCGCA	1866063	1205548	Yes
SISSAQ12X03G_0039	Diploid	TAGTAT	1808693	1219474	Yes
SISSAQ12X03G_0006	Diploid	GGACTT	1879529	1243091	Yes
SISSAQ12X03G_0007	Diploid	TAAGCT	2009004	1255179	Yes
SISSAQ12X03G_0083	Diploid	CCGCAT	1763010	1412054	Yes
SISSAQ12X03G_0015	Diploid	TAATTC	2309771	1414278	Yes
SISSAQ12X03G_0066	Diploid	ATAGTA	2027450	1469995	Yes
SISSAQ12X03G_0055	Diploid	TCAAAG	2211056	1560982	No
SISSAQ12X03G_0059	Diploid	CCCGGT	2204743	1647765	No
SISSAQ12X03G_0052	Diploid	CTACAG	2479627	1650436	No
SISSAQ12X03G_0014	Diploid	GGCAAG	2421984	1652405	No
SISSAQ12X03G_0078	Diploid	GTGCCT	2133015	1654724	No
SISSAQ12X03G_0065	Diploid	ACAGCG	2244186	1733381	No
SISSAQ12X03G_0008	Diploid	TCTTCT	2728753	1782222	No
SISSAQ12X03G_0086	Diploid	GTGTAA	2365105	1853750	No
SISSAQ12X03G_0001	Diploid	AAACGG	2933658	1876248	No
SISSAQ12X03G_0046	Diploid	GGTTTG	2678588	1905504	No
SISSAQ12X03G_0068	Diploid	CTGCGA	2376959	1929445	No
SISSAQ12X03G_0073	Diploid	ACATAC	2854175	2039150	No
SISSAQ12X03G_0081	Diploid	ACCATG	2640014	2044696	No
SISSAQ12X03G_0042	Diploid	AGGTGT	3056856	2161717	No

SISSAQ12X03G_0013	Diploid	GAACCA	3330881	2215694	No
SISSAQ12X03G_0051	Diploid	CCATTT	3009640	2220926	No
SISSAQ12X03G_0033	Diploid	AAGCTA	3055985	2250476	No
SISSAQ12X03G_0082	Diploid	ATGCAC	2865774	2288716	No
SISSAQ12X03G_0016	Diploid	TGAACC	3519094	2288838	No
SISSAQ12X03G_0003	Diploid	ATTCCG	3249675	2293647	No
SISSAQ12X03G_0085	Diploid	GCGCTG	2823027	2308992	No
SISSAQ12X03G_0070	Diploid	GTCATC	2853477	2312704	No
SISSAQ12X03G_0067	Diploid	CCCTAA	2885781	2316256	No
SISSAQ12X03G_0090	Diploid	ATGTTG	3121980	2391536	No
SISSAQ12X03G_0060	Diploid	CTCGCC	3176130	2508603	No
SISSAQ12X03G_0005	Diploid	GAAATG	3844306	2513394	No
SISSAQ12X03G_0010	Diploid	ACTGGC	3677787	2513686	No
SISSAQ12X03G_0012	Diploid	CGCAGA	3624389	2536518	No
SISSAQ12X03G_0043	Diploid	CATACT	3584395	2543547	No
SISSAQ12X03G_0077	Diploid	GCGACC	3065185	2554427	No
SISSAQ12X03G_0076	Diploid	CTGGTT	3197555	2574112	No
SISSAQ12X03G_0088	Diploid	TTTAAT	3278967	2610584	No
SISSAQ12X03G_0029	Diploid	GACTCT	3900459	2624828	No
SISSAQ12X03G_0063	Diploid	TCCTGC	3501080	2643892	No
SISSAQ12X03G_0096	Diploid	TTTGTC	3305497	2664060	No
SISSAQ12X03G_0004	Diploid	CGAGGC	3874578	2714132	No
SISSAQ12X03G_0036	Diploid	CGTCTA	3536215	2720279	No
SISSAQ12X03G_0045	Diploid	GATCGT	3572836	2814035	No
SISSAQ12X03G_0075	Diploid	CCGAGG	3454474	2861569	No
SISSAQ12X03G_0050	Diploid	AGTAGG	3933543	2903682	No
SISSAQ12X03G_0087	Diploid	TCGGAC	3543751	2953320	No
SISSAQ12X03G_0061	Diploid	GCATGG	3879811	2963191	No
SISSAQ12X03G_0056	Diploid	TTATGA	4141134	2985877	No
SISSAQ12X03G_0034	Diploid	AGGGTC	3901685	3022465	No
SISSAQ12X03G_0072	Diploid	TTCTAG	3647368	3027041	No
SISSAQ12X03G_0092	Diploid	CTTTGC	3784843	3089603	No
SISSAQ12X03G_0062	Diploid	GTATCC	4055968	3132625	No
SISSAQ12X03G_0093	Diploid	GCTCAA	3817872	3169262	No
SISSAQ12X03G_0091	Diploid	CCTAAC	3936395	3241607	No
SISSAQ12X03G_0038	Diploid	GGTACA	4177973	3262012	No
SISSAQ12X03G_0079	Diploid	TCGCCA	3940295	3313525	No
SISSAQ12X03G_0094	Diploid	GTTGGA	4019145	3319122	No
SISSAQ12X03G_0089	Diploid	ACCCCC	4137078	3341016	No
SISSAQ12X03G_0054	Diploid	GTAAGT	4461160	3439564	No
SISSAQ12X03G_0071	Diploid	TCGATT	4102807	3507928	No
SISSAQ12X03G_0018	Diploid	AGCCAT	5053655	3522228	No
SISSAQ12X03G_0031	Diploid	TACGGG	5228027	3621238	No
SISSAQ12X03G_0023	Diploid	TACACA	5731146	3628053	No

SISSAQ12X03G_0011	Diploid	CAAAAA	5698776	3635387	No
SISSAQ12X03G_0019	Diploid	CAATCG	5389309	3805788	No
SISSAQ12X03G_0053	Diploid	GCAGAT	4833582	3827127	No
SISSAQ12X03G_0064	Diploid	TTCCGT	4897913	3851508	No
SISSAQ12X03G_0030	Diploid	GGGGCG	5440455	3914714	No
SISSAQ12X03G_0080	Diploid	TTGAGC	4695716	3932367	No
SISSAQ12X03G_0022	Diploid	GGGCGC	5372161	4011058	No
SISSAQ12X03G_0032	Diploid	TGCCCG	5896229	4170112	No
SISSAQ12X03G_0024	Diploid	TGACAA	6259407	4208364	No
SISSAQ12X03G_0041	Diploid	AATATC	5508336	4211915	No
SISSAQ12X03G_0035	Diploid	CAGGCA	5706813	4314728	No
SISSAQ12X03G_0020	Diploid	CGCGTG	5996362	4362744	No
SISSAQ12X03G_0040	Diploid	TGCTTA	5711878	4381971	No
SISSAQ12X03G_0044	Diploid	CGTGAT	5604149	4431299	No
SISSAQ12X03G_0084	Diploid	CTTATG	5370280	4483972	No
SISSAQ12X03G_0028	Diploid	CGGTCC	6809724	5039216	No
SISSAQ12X03G_0027	Diploid	CACCTC	7873624	5449126	No
SISSAQ12X03G_0095	Diploid	TCTCGG	7013123	5975439	No
SISSAQ12X03G_0047	Diploid	TATCAC	7767702	6062053	No

Table S3. Linkage map with the following information for each locus; marker name, supported segregation model inferred from the method of Waples *et al.* (2015), duplication status, LG (So), LG arm, position (cM), *y* value, RAD-tag sequence.

Available for download as an Excel file at www.g3journal.org/lookup/suppl/doi:10.1534/g3.115.020222/-/DC1

Table S4 Interference results from the CODA analysis with estimates and (±95% CI) for v and *p* for all LGs. Chromosome type denotes telocentric (T) or metacentric (M) designations of each LG.

LG (So)	Chromosome type	v (95% CI)	p (95% CI)
1	T	12.89 (10.82)	0.08 (0.12)
2	M	6.58 (3.62)	0.04 (0.06)
3	M	8.51 (4.16)	0.03 (0.05)
4	M	6.31 (8.16)	0.16 (0.21)
5	M	3.69 (1.24)	0 (0.16)
6	M	4.4 (1.37)	0 (0.08)
7	M	9.57 (6.09)	0.07 (0.08)
8	M	8.29 (4.9)	0.08 (0.07)
9A_(X_2)	T	NA	NA
9B_(X_1)	T	NA	NA
10	M	6.33 (2.25)	0 (0.13)
11	M	5.81 (3.6)	0.04 (0.09)
12	M	10.52 (6.16)	0.12 (0.08)
13	M	4.53 (2.07)	0.04 (0.07)
14	M	10.65 (7.42)	0.13 (0.09)
15	M	5.1 (2.04)	0.05 (0.06)
16	T	14.54 (17.04)	0.14 (0.14)
17	T	3.06 (1.27)	0 (0.38)
18A	M (arm1)	NA	NA
18B	M (arm2)	NA	NA
19	M	9.16 (3.75)	0.02 (0.03)
20	M	13.91 (5.29)	0.05 (0.04)
21	M	5.74 (1.96)	0 (0.06)
22	M	7.31 (2.51)	0.05 (0.04)
23	M	6.36 (2.22)	0 (0.19)
24	M	6.72 (2.25)	0 (0.06)
25	T	NA	NA
26	T	NA	NA
27	M	3.76 (1.16)	0 (0.13)
28	M	1.61 (0.89)	0 (1.11)

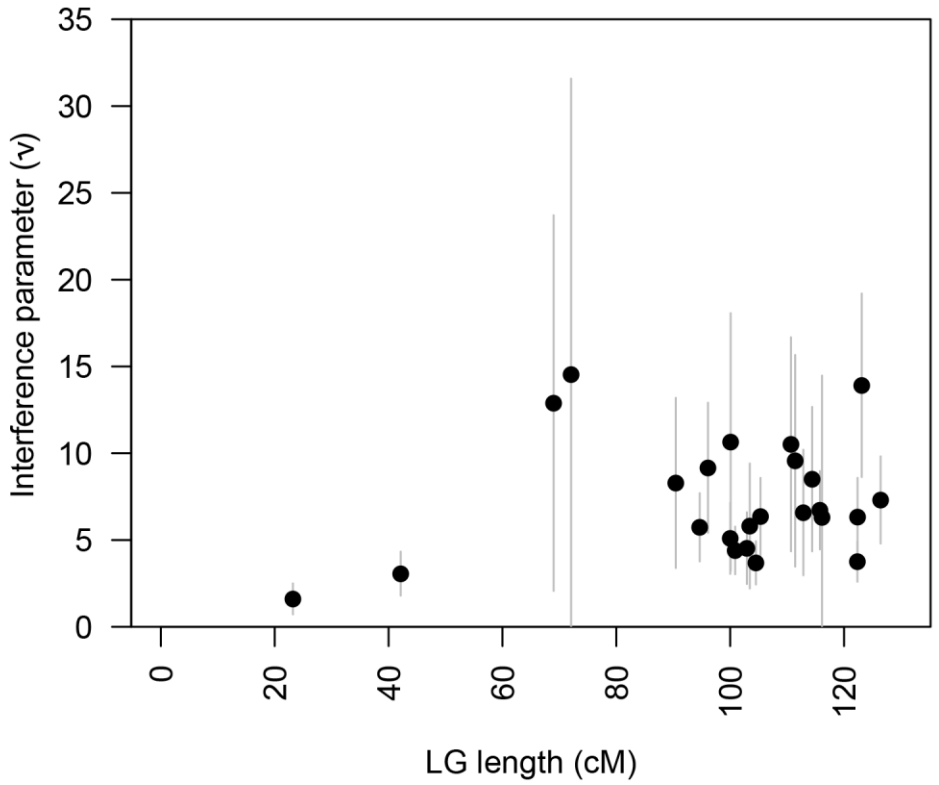

Figure S1 Estimates of the interference parameter (ν). Values of ν are plotted against map length of the linkage groups where estimates of ν were obtained. Vertical grey lines show the 95% CI around each ν estimate.
