# Supplementary material for: Linkage Mapping Reveals Strong Chiasma Interference in Sockeye Salmon: Implications for Interpreting Genomic Data
Source: G3 (Bethesda). 2015 Sep 18;5(11):2463–73. doi: 10.1534/g3.115.020222 (PMC4632065; doi:10.1534/g3.115.020222)
Supplement: Supporting Information [file supp_g3.115.020222_FileS1.pdf]

File S1

Supporting Materials and Methods

Restriction site associated DNA (RAD) sequencing, SNP discovery, and genotyping

We identified and resolved duplicated loci, here defined as assemblies representing two distinct genomic locations, using the allelic segregation patterns in the haploid offspring. In *Stacks* (Catchen *et al.* 2011; Catchen *et al.* 2013) and during other approaches to discover and genotype loci *de novo*, sequences are aligned, and similar sequences are assembled into loci. In cases of sequence, gene, or genome duplication, paralogous sequences can erroneously be assigned to the same locus, here termed duplicated loci. We follow the approach of Waples *et al.* (2015) and attempt to resolve and include individual loci from these confounded assemblies on the linkage map.

For the sockeye salmon (*Oncorhynchus nerka*) parent and each haploid offspring, we considered stacks of minimum two identical reads as representing unique alleles and allowed four unique stacks (the maximum number of expected alleles for a duplicated locus) with up to four nucleotide mismatches for building *de novo* loci. In order to resolve duplicated loci based on segregation within the mapping family, we disabled the deleveraging algorithm within Stacks (Catchen *et al.* 2013), which is designed to resolve them based on sequence similarity. Then we created a catalog from the female parent and assigned genotypes to haploid offspring by matching to RAD loci in the parental catalog.

We identified duplicated loci as *Stacks* assemblies with segregation patterns consistent with two confounded disomic loci, considering the possibility of full allelic identity (i.e. isoloci). For inclusion on a linkage map, a locus must be heterozygous (segregating) in the parent. Identified duplicated loci were resolved into zero, one, or two separate loci eligible for inclusion on the linkage map, based on variability in the female parent and the ability to infer segregation in the offspring (see Waples *et al.* 2015).

At this point, genotypes from 5'-nuclease assays and RAD loci were combined into a single data set for creation of a linkage map. Loci with missing genotypes in more than 25% of the haploid

offspring or that showed significant segregation distortion ($P<0.05$ after a false discovery rate correction) were removed prior to linkage map construction (Waples *et al.* 2015).

Comparative mapping, annotation, and sex chromosomes

Here, we used both intra- and interspecific comparisons to effectively validate LG assemblies, maintain nomenclature of LGs and identify sex chromosomes.

Annotation of linkage groups

We compared the linkage map with the existing male and female maps for anadromous sockeye salmon developed with a similar RAD sequencing approach (Everett *et al.* 2012). We used the BLASTN software (BLAST version 2.2.28; Altschul *et al.* 1990) to align a fasta file with sequences for each locus on our linkage map. The first 59 bp of each sequence were considered to match another fasta file with 59 bp allelic sequences for each locus on the map by Everett *et al.* (2012). We allowed a maximum of two mismatches and considered the alignment with the lowest e-value as signifying homology between the two sockeye salmon maps. Matching RAD loci were used to identify and annotate LGs according to Everett *et al.* (2012).

Identification of sex chromosomes

We aligned sequences for mapped loci to a linkage map for rainbow trout (*O. mykiss*) generated with a similar RAD-sequencing approach (Miller *et al.* 2012) to identify the X_1 and X_2 sex chromosomes based on known synteny. Sockeye salmon have a unique X_1X_2Y sex chromosome system, where a single copy of the metacentric Neo-Y chromosome is present only in males whereas females have two copies each of the two acrocentric chromosomes X_1 and X_2. (Thorgaard 1978; Faber-Hammond *et al.* 2012).The sockeye salmon ancestral sex chromosome X_1 is acrocentric and known to share homology with chromosome *Omy*08 in rainbow trout, whereas the acrocentric X_2 share homology with *Omy*02.

The rainbow trout map has been paired with corresponding physical chromosomes for rainbow trout (Palti *et al.* 2011) which allowed for the identification of sockeye salmon linkage groups that share synteny with *Omy*08 and *Omy*02. We used the BLASTN software to align a fasta file with RAD-tag sequences from our map to all 60 bp mapped RAD-tags on the rainbow trout linkage map (Miller *et al.* 2012). Compared to the intraspecific comparison described above, we allowed sequences to be

more diverged between the two species; we allowed up to two mismatches, alignment lengths of at least 58 of 60 bp, and a maximum of two gaps as signifying orthologous loci in rainbow trout. The alignment with the lowest e-value was retained.

The ancestral sex chromosome (X_1) was identified as LG9B and the acrocentric autosome (X_2) was identified as LG9A on our map. This comports with the result that LG9 represents the metacentric Neo-Y chromosome in the male sockeye salmon map of Everett *et al.* (2012). Furthermore, most LGs aligned to either a single or two different LGs between the two species (Suppl. Table S1). Conserved synteny has been observed among chromosome arms coupled with a range of Robertsonian rearrangements after the two species diverged (see discussion in Naish *et al.* 2013). In a few cases, loci from a single LG aligned to more than two LGs in the other species; this may be explained by single erroneous alignments since no more than two LGs were supported by more than one locus from the corresponding sockeye salmon LG (Table S1).

References

Altschul, S. F., W. Gish, W. Miller, E. W. Myers and D. J. Lipman, 1990 Basic local alignment search tool. J. Mol. Biol. 215**:** 403-410.

Catchen, J., P. A. Hohenlohe, S. Bassham, A. Amores and W. A. Cresko, 2013 Stacks: an analysis tool set for population genomics. Mol. Ecol. 22**:** 3124-3140.

Catchen, J. M., A. Amores, P. Hohenlohe, W. Cresko and J. H. Postlethwait, 2011 Stacks: Building and genotyping loci de novo from short-read sequences. G3 1**:** 171-182.

Everett, M. V., M. R. Miller and J. E. Seeb, 2012 Meiotic maps of sockeye salmon derived from massively parallel DNA sequencing. BMC Genomics 13**:** 521.

Faber-Hammond, J., R. B. Phillips and L. K. Park, 2012 The sockeye salmon neo-Y chromosome is a fusion between linkage lroups orthologous to the coho Y chromosome and the long arm of rainbow trout chromosome 2. Cytogenet Genome Res 136**:** 69-74.

Miller, M. R., J. P. Brunelli, P. A. Wheeler, S. X. Liu, C. E. Rexroad *et al.*, 2012 A conserved haplotype controls parallel adaptation in geographically distant salmonid populations. Mol. Ecol. 21**:** 237-249.

Naish, K. A., R. B. Phillips, M. S. O. Brieuc, L. R. Newton, A. E. Elz *et al.*, 2013 Comparative genome mapping between Chinook salmon (*Oncorhynchus tshawytscha*) and rainbow trout (*O. mykiss*) based on homologous microsatellite loci. G3 3**:** 2281-2288.

Palti, Y., C. Genet, M.-C. Luo, A. Charlet, G. Gao *et al.*, 2011 A first generation integrated map of the rainbow trout genome. BMC Genomics 12**:** 180.

Thorgaard, G. H., 1978 Sex-chromosomes in sockeye salmon - Y-Autosome fusion. Can. J. Genet. Cytol. 20**:** 349-354.

Waples, R. K., L. W. Seeb and J. E. Seeb, 2015 Linkage mapping with paralogs exposes regions of residual tetrasomic inheritance in chum salmon (*Oncorhynchus keta*). Mol. Ecol. Resour. doi: 10.1111/1755-0998.12394.
