# Supplementary material for: Linkage Mapping Reveals Strong Chiasma Interference in Sockeye Salmon: Implications for Interpreting Genomic Data
Source: G3 (Bethesda). 2015 Sep 18;5(11):2463–73. doi: 10.1534/g3.115.020222 (PMC4632065; doi:10.1534/g3.115.020222)
Supplement: Supporting Information [file supp_g3.115.020222_FigureS1.pdf]

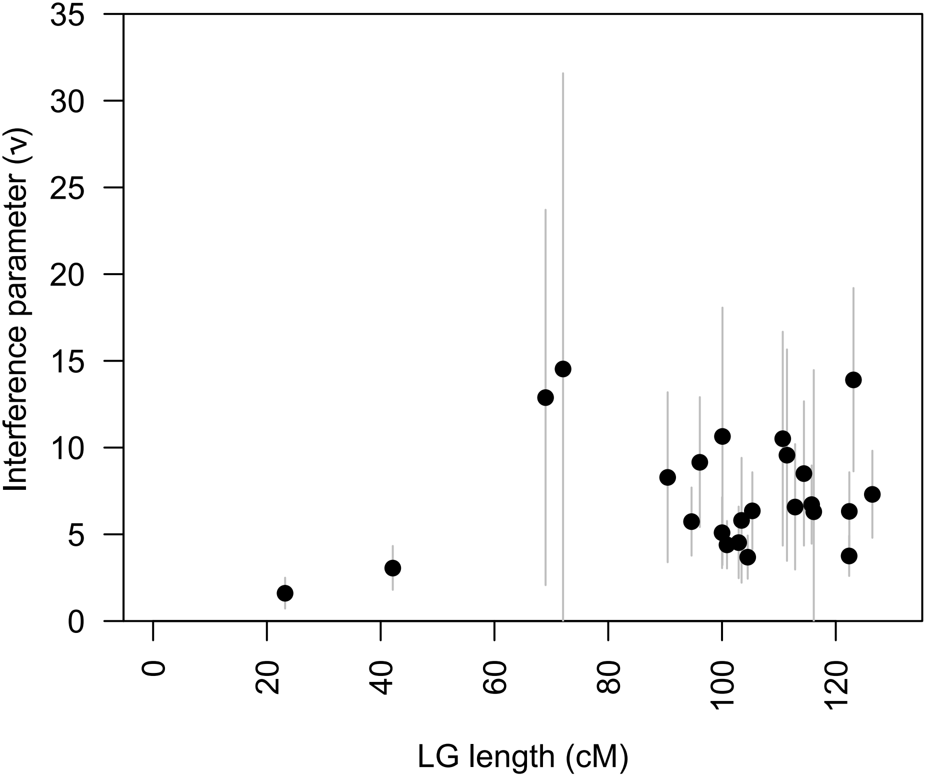

Interference parameter (ν)
35
30
25
20
15
10
5
0
0
20
40
60
80
100
120
LG length (cM)

Figure S1 Estimates of the interference parameter (ν). Values of ν are plotted against map length of the linkage groups where estimates of ν were obtained. Vertical grey lines show the 95% CI around each ν estimate.
