# Supplementary material for: Linkage Mapping Reveals Strong Chiasma Interference in Sockeye Salmon: Implications for Interpreting Genomic Data
Source: G3 (Bethesda). 2015 Sep 18;5(11):2463–73. doi: 10.1534/g3.115.020222 (PMC4632065; doi:10.1534/g3.115.020222)
Supplement: Supporting Information [file supp_g3.115.020222_TableS1.pdf]

Table S1 The table presents syntenic relationships among chromosome arms between linkage maps for sockeye salmon (this study) and rainbow trout (Miller *et al.* 2012). The number of loci supporting each syntenic pair is given.

Sockeye salmon LG (This study)	Number of loci supporting synteny	Rainbow trout LG (Miller et al. 2012)	Rainbow trout Chr. (Palti et al. 2011)
**1**	5	WS02	Omy22
**1**	1	WS16	Omy27
**2**	4	WS25	Omy07
**3**	1	WS04	Omy09
**3**	2	WS06	Omy03
**4**	8	WS03	Omy05
**4**	2	WS19	Omy18
**5**	1	WS19	Omy18
**5**	4	WS24	Omy25
**6**	1	WS12	Omy15
**7**	3	WS04	Omy09
**7**	1	WS10	Omy14
**7**	1	WS12	Omy15
**8**	1	WS17	Omy24
**8**	1	WS26	Omy21
**9A_(X_2)**	4	WS18	Omy02
**9B_(X_1)**	2	WS05	Omy08
**10**	2	WS14	Omy10
**10**	1	WS24	Omy25
**11**	1	WS03	Omy05
**11**	3	WS16	Omy27
**12**	2	WS04	Omy09
**12**	4	WS24	Omy25
**13**	2	WS01	Omy04
**13**	1	WS06	Omy03
**13**	2	WS07	Omy11

**14**	1	WS17	Omy24
**14**	1	WS18	Omy02
**15**	4	WS23	Omy17
**16**	1	WS15	Omy20
**16**	1	WS20	Omy01
**17**	3	WS01	Omy04
**18A**	0	N/A	N/A
**18B**	1	WS22	Omy19
**19**	3	WS11	OmySex
**20**	3	WS08	Omy16
**20**	1	WS09	Omy28
**20**	1	WS23	Omy17
**21**	0	N/A	N/A
**22**	5	WS07	Omy11
**22**	2	WS21	Omy12
**23**	3	WS08	Omy16
**23**	1	WS12	Omy15
**23**	1	WS24	Omy25
**24**	6	WS03	Omy05
**24**	1	WS05	Omy08
**24**	1	WS18	Omy02
**25**	1	WS14	Omy10
**25**	5	WS20	Omy01
**26**	1	WS08	Omy16
**27**	1	WS13	Omy06
**28**	0	N/A	N/A
