# Supplementary material for: Linkage Mapping Reveals Strong Chiasma Interference in Sockeye Salmon: Implications for Interpreting Genomic Data
Source: G3 (Bethesda). 2015 Sep 18;5(11):2463–73. doi: 10.1534/g3.115.020222 (PMC4632065; doi:10.1534/g3.115.020222)
Supplement: Supporting Information [file supp_g3.115.020222_TableS4.pdf]

Table S4 Interference results from the CODA analysis with estimates and (±95% CI) for v and *p* for all LGs. Chromosome type denotes telocentric (T) or metacentric (M) designations of each LG.

LG (So)	Chromosome type	v (95% CI)	p (95% CI)
1	T	12.89 (10.82)	0.08 (0.12)
2	M	6.58 (3.62)	0.04 (0.06)
3	M	8.51 (4.16)	0.03 (0.05)
4	M	6.31 (8.16)	0.16 (0.21)
5	M	3.69 (1.24)	0 (0.16)
6	M	4.4 (1.37)	0 (0.08)
7	M	9.57 (6.09)	0.07 (0.08)
8	M	8.29 (4.9)	0.08 (0.07)
9A_(X_2)	T	NA	NA
9B_(X_1)	T	NA	NA
10	M	6.33 (2.25)	0 (0.13)
11	M	5.81 (3.6)	0.04 (0.09)
12	M	10.52 (6.16)	0.12 (0.08)
13	M	4.53 (2.07)	0.04 (0.07)
14	M	10.65 (7.42)	0.13 (0.09)
15	M	5.1 (2.04)	0.05 (0.06)
16	T	14.54 (17.04)	0.14 (0.14)
17	T	3.06 (1.27)	0 (0.38)
18A	M (arm1)	NA	NA
18B	M (arm2)	NA	NA
19	M	9.16 (3.75)	0.02 (0.03)
20	M	13.91 (5.29)	0.05 (0.04)
21	M	5.74 (1.96)	0 (0.06)
22	M	7.31 (2.51)	0.05 (0.04)
23	M	6.36 (2.22)	0 (0.19)
24	M	6.72 (2.25)	0 (0.06)
25	T	NA	NA
26	T	NA	NA
27	M	3.76 (1.16)	0 (0.13)
28	M	1.61 (0.89)	0 (1.11)
